# PGE_2_ upregulates gene expression of dual oxidase in a lepidopteran insect midgut via cAMP signalling pathway

**DOI:** 10.1098/rsob.200197

**Published:** 2020-10-21

**Authors:** Seyedeh Minoo Sajjadian, Yonggyun Kim

**Affiliations:** Department of Plant Medicals, College of Life Sciences, Andong National University, Andong 36729, Korea

**Keywords:** dual oxidase, prostaglandin, cAMP, reactive oxygen species, gut immunity, *Spodoptera exigua*

## Abstract

In insect midgut, prostaglandins (PGs) play a crucial role in defending bacterial and malarial pathogens. However, little is known about the PG signalling pathway in the midgut. A dual oxidase (*Se-Duox*) with presumed function of catalysing reactive oxygen species (ROS) production in the midgut was identified in beet armyworm, *Spodoptera exigua*. *Se-Duox* was expressed in all developmental stages, exhibiting relatively high expression levels in the midgut of late larval instars. *Se-Duox* expression was upregulated upon bacterial challenge. RNA interference (RNAi) of *Se-Duox* expression significantly suppressed ROS levels in the midgut lumen. The suppression of ROS levels increased insecticidal activity of *Serratia marcescens* after oral infection. Interestingly, treatment with a PLA_2_ inhibitor prevented the induction of *Se-Duox* expression in response to bacterial challenge. On the other hand, addition of its catalytic product rescued the induction of *Se-Duox* expression. Especially, PG synthesis inhibitor significantly suppressed *Se-Duox* expression, while the addition of PGE_2_ or PGD_2_ rescued the inhibition. Subsequent PG signals involved cAMP and downstream components because specific inhibitors of cAMP signal components such as adenylate cyclase (AC) and protein kinase A (PKA) significantly inhibited *Se-Duox* expression. Indeed, addition of a cAMP analogue stimulated *Se-Duox* expression in the midgut. Furthermore, individual RNAi specific to PGE_2_ receptor (a trimeric G-protein subunit), AC, PKA or cAMP-responsive element-binding protein resulted in suppression of *Se-Duox* expression. These results suggest that PGs can activate midgut immunity via cAMP signalling pathway by inducing *Se-Duox* expression along with increased ROS levels.

## 1. Introduction

The insect gut is usually exposed to various microbes. The insect foregut and hindgut are originated from embryonic ectoderm. They can protect insects from pathogen infection through their cuticle linings. The midgut lacking a cuticle barrier is known to be relatively susceptible to various microbial pathogens such as bacteria, viruses, nematodes and protozoa [1]. Instead of a cuticle layer, the midgut in many insects possesses a mucus layer and a peritrophic matrix that can act as physical barriers against microbial infections [2]. In addition, insect midguts possess commensal or mutualistic microbes required for assisting digestion, supplementing essential nutrients, detoxifying xenochemicals or defending against other pathogens [3].

In addition to these protective defence lines, insect midguts exhibit direct antimicrobial activities by producing reactive oxygen species (ROS) and antimicrobial peptides (AMPs) [4]. However, these chemical defences should be tightly regulated so that beneficial microbes could be protected in insect midguts, while pathogens could be selectively removed [5]. For example, peptidoglycans derived from invading bacterial pathogens can trigger AMP expression [6]. However, Caudal, a homeobox protein, can selectively repress the expression of IMD/NF-kB-dependent AMP genes to protect beneficial symbiotic bacteria in the midgut [7]. In addition, ROS are specifically produced by pathogens [8–10].

ROS are produced in insect midguts by two different kinds of enzymes: NADPH-dependent oxidase (Nox) and dual oxidase (Duox) [11]. Inducing activities of these enzymes is crucial for the regulation of toxic ROS production. *Nox* expression can be induced by lactic acid produced by anaerobic metabolism of pathogens [12], while *Duox* expression is regulated by uracil released from pathogens in *Drosophila* [10]. However, it remains unclear how pathogen-derived factors can activate oxidases to upregulate ROS levels.

Eicosanoids are a group of oxygenated C20 polyunsaturated fatty acids that can mediate various physiological processes including immune responses in metazoan animals [13]. In *Spodoptera exigua*, a lepidopteran insect, Nox gene expression and subsequent ROS production in haemocytes are regulated by eicosanoids [14]. In the midgut of *Plutella xylostella*, another lepidopteran insect, Duox gene expression and subsequent ROS production that are essential for defending against bacterial pathogens are inhibited by treatment with eicosanoid biosynthesis inhibitors [11]. These results suggest that eicosanoids can mediate ROS production by activating Nox or Duox in these lepidopteran insects. Among eicosanoids, PGs play crucial role in mediating various immune responses in midguts of lepidopteran and dipteran insects [11,15]. In particular, several PGs along with PGE_2_ receptor (PGR) and its downstream signal components have been found in *S. exigua* [16]. Thus, the hypothesis of this study was that PGE_2_ could mediate ROS production by inducing *Duox* expression in *S. exigua* through cAMP signalling pathway.

## 2. Results

### 2.1. Identification of *Se-Duox* and domain analysis

**Figure 1.**
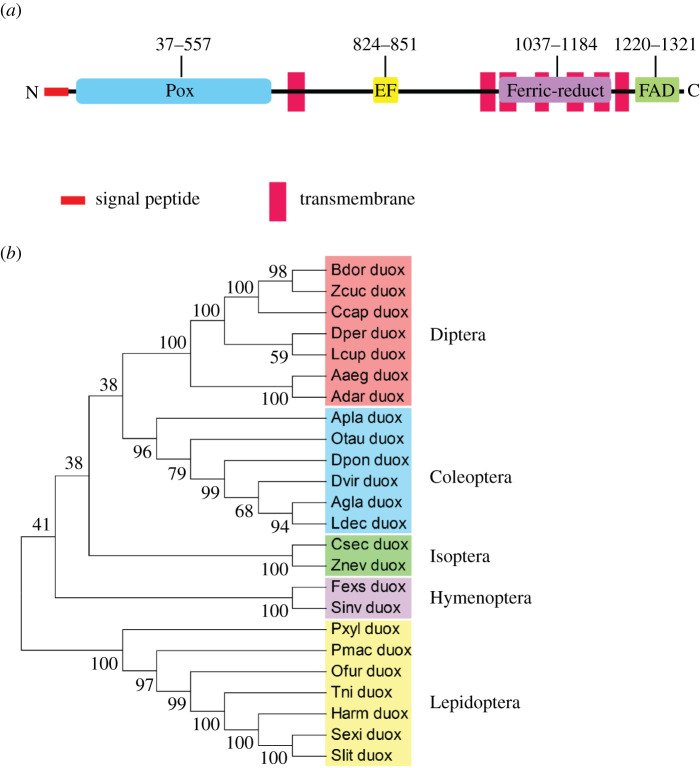
Molecular characterization of *Se-Duox*. (*a*) Functional domain analysis of *Se-Duox*. Domains were predicted using HMMER (https://www.ebi.ac.uk) and Pfam (http://pfam.xfam.org). Predicted domains include ‘Pox’ for peroxidase, ‘EF’ for calcium-binding EF hand, ‘Ferric-reduct’ for ferric chelate reductase, ‘FAD’ for FAD-binding domain and ‘NAD’ for NAD-binding domain. (*b*) Phylogenetic analysis of *Se-Duox* with other insect dual oxidases based on their amino acid sequences. The tree was generated by the neighbour-joining method using MEGA 6.0. Bootstrapping values were obtained with 1000 repetitions to support branch and clustering. Amino acid sequences were retrieved from GenBank. Accession numbers of genes are shown in electronic supplementary material, table S2.

Transcriptome of *S. exigua* was interrogated with Duox gene of *Drosophila melanogaster*. A highly matched sequence (*Se-Duox*) encoding 1594 amino acid residues (figure 1) was found. It was predicted to be a transmembrane protein because of the presence of a signal peptide and transmembrane domains (figure 1*a*). It contains two oxidase domains (peroxidase at the N terminus and FAD-linked oxidase at C-terminus). When *Se-Duox* was phylogenetically analysed with several insect Duox genes, it formed a monophyletic cluster with other lepidopteran orthologues (figure 1*b*).

### 2.2. Inducible expression of *Se-Duox* upon bacterial challenge

**Figure 2.**
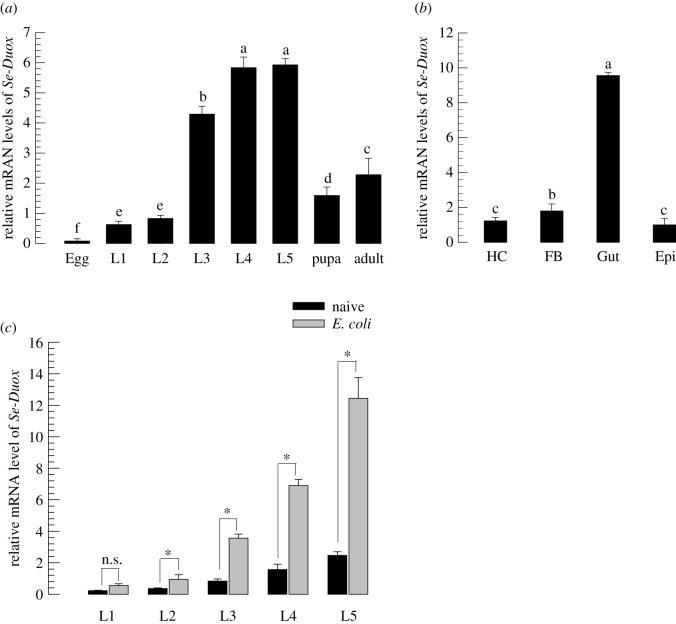
Expression profile of *Se-Duox*. (*a*) Expression patterns of *Se-Duox* in different developmental stages: egg, first to fifth instar larvae (‘L1–L5’), pupa and adult. (*b*) Expression patterns of *Se-Duox* in indicated tissues of L5D2 larvae, including haemocyte (HC), fat body (FB), midgut (Gut) and epidermis (Epi). (*c*) Induction of *Se-Duox* expression in response to bacterial challenge. L1–L5 larvae were fed with *E. coli* (2 × 10^4^)-treated artificial diet for 8 h. Midguts were then dissected to study expression patterns of *Se-Duox*. A ribosomal gene, RL32, was used as a reference gene. Each treatment was replicated three times with independent tissue preparations. Different letters above standard deviation bars indicate significant differences among means at Type I error = 0.05 (LSD test).

*Se-Duox* was expressed in all developmental stages of *S. exigua* (figure 2*a*). It was highly expressed in late larval instars. In the last larval instar (L5), *Se-Duox* was highly expressed in the midgut (figure 2*b*). Bacterial challenge significantly (*p* < 0.05) increased its gene expression levels in all larval instars (figure 2*c*).

### 2.3. RNA interference of *Se-Duox* suppresses ROS level and enhances bacterial pathogenicity

**Figure 3.**
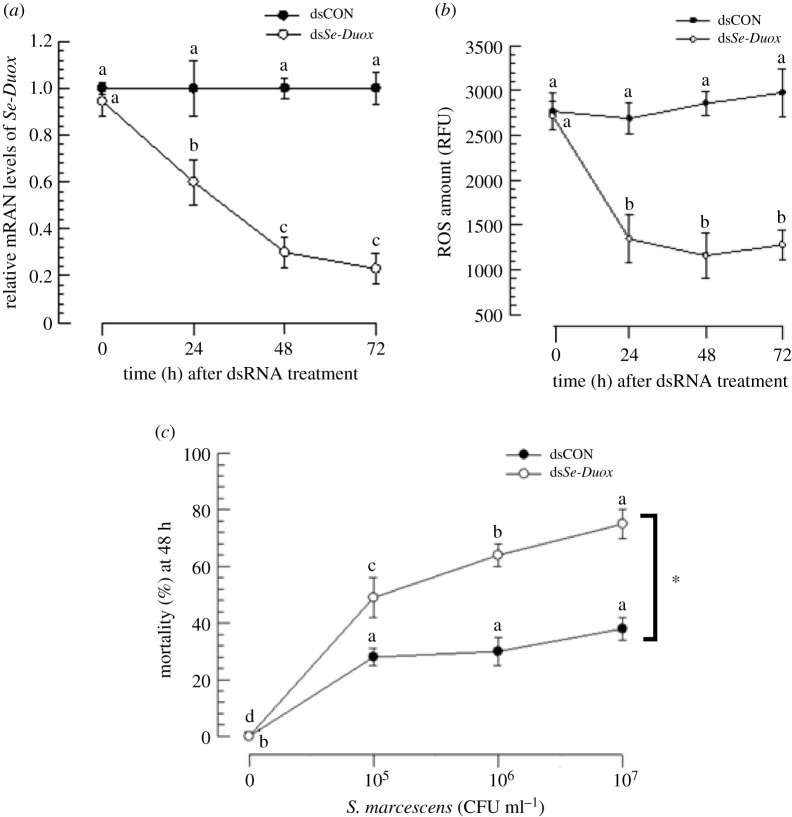
RNAi of *Se-Duox* expression and subsequent influence on ROS level and larval susceptibility to *S. marcescens*. (*a*) Effects of RNAi on *Se-Duox* expression at different time points in midguts of *S. exigua* larvae (L5). One microgram of gene-specific dsRNA (ds*Se-Duox*) was injected into each larva. CpBV302, a viral gene, was used to generate control dsRNA (dsCON). (*b*) Inhibitory effects of RNAi specific to *Se-Duox* expression on total ROS levels in the midgut. To induce Duox expression and ROS, after dsRNA treatments, larvae were fed with *E. coli* (2 × 10^4^ cells) treated artificial diet for 12 h. (*c*) Effects of *Se-Duox* RNAi on pathogenicity of *S. marcescens* against L4 larvae of *S. exigua*. RNAi-treated larvae were exposed to different concentrations of *S. marcescens*. Mortality was recorded at 48 h post feeding. Each treatment was replicated three times. Each replication used 10 larvae. Different letters above standard deviation bars indicate significant difference among means in each treatment and control at Type I error = 0.05 (LSD test). Asterisk indicates the statistical difference between control and treatment at 10^7^ CFU ml^−1^ dose.

Injection of dsRNA specific to *Se-Duox* to L5 larvae significantly (*p* < 0.05) suppressed its expression. Such RNA interference (RNAi) efficiency was maintained for at least 72 h PI (figure 3*a*). Along with decrease in *Se-Duox* expression, ROS levels in the gut lumen were also significantly (*p* < 0.05) reduced (figure 3*b*). Such a decrease in ROS levels increased insecticidal activity of *S. marcescens* after oral administration (figure 3*c*).

### 2.4. PGs activate *Se-Duox* expression

**Figure 4.**
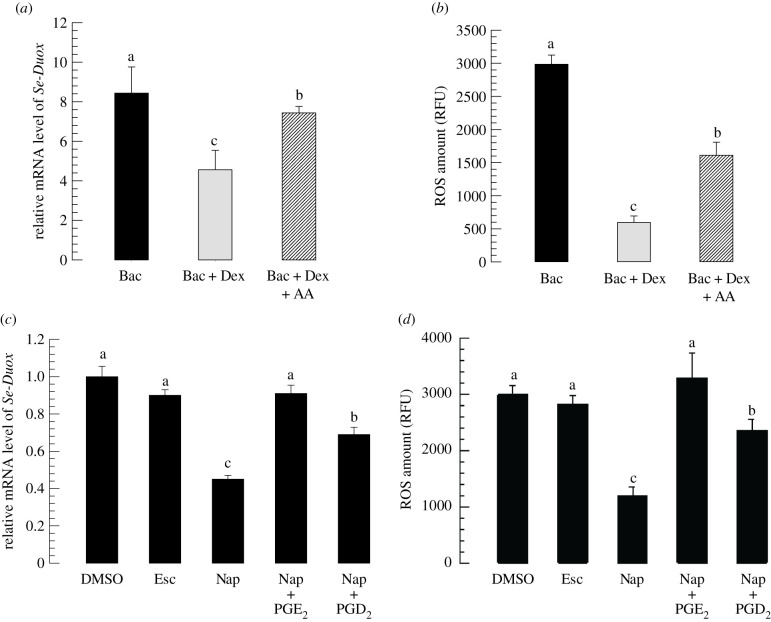
Effects of PLA_2_ inhibitor and COX and LOX inhibitors on expression levels of *Se-Duox*. (*a*) Inhibitory effect of a PLA_2_ inhibitor, dexamethasone (DEX), on *Se-Duox* expression in L5 larvae. For bacterial challenge, larvae were fed with *E. coli* (2 × 10^4^ cells/larva) treated artificial diet for 12 h. (*b*) Effects of DEX on ROS levels in gut lumen. To induce ROS, *E. coli* (2 × 10^4^ cells)-treated artificial diet was fed to larvae at 8 h post-injection of DEX. At 12 h after bacterial treatment, intestinal ROS levels were measured. (*c*) Influence of naproxene (‘Nap’, a COX inhibitor) and esculetin (‘Esc’, a LOX inhibitor) on expression of *Se-Duox*. (*d*) Effects of Nap and Esc on ROS levels in gut lumen. Each inhibitor was injected into larvae at a final concentration of 10 µg per larva. At 8 h post-injection of inhibitor, *E. coli* (2 × 10^4^ cells)-treated artificial diet were fed to larvae to induce *Se-Duox* expression and ROS. To rescue *Se-Duox* expression and ROS production, PGD_2_ or PGE_2_ was injected at 1 µg/larva. Each treatment was replicated three times. Each replication used 15 larvae. Different letters indicate significant differences among means at Type I error = 0.05 (LSD test).

Next, the effect of eicosanoids on induction of *Se-Duox* expression upon bacterial challenge was analysed. PLA_2_ activity was significantly (*p* < 0.05) inhibited by its specific inhibitor dexamethasone (Dex). Bacterial induction of *Se-Duox* expression was reduced by such inhibitor treatment (figure 4*a*). However, an addition of arachidonic acid (‘AA’, a catalytic product of PLA_2_) to Dex-treated larvae significantly (*p* < 0.05) rescued such inhibition of *Se-Duox* expression. ROS levels in gut lumen were highly dependent on *Se-Duox* expression levels. Dex treatment suppressed ROS levels, whereas the addition of AA rescued ROS levels suppressed by Dex treatment (figure 4*b*).

Modulation of *Se-Duox* expression by Dex or AA treatment suggested that *Se-Duox* expression could be controlled by eicosanoids. To clarify the types of eicosanoids that could modulate *Se-Duox* expression, esculetin (Esc) known to inhibit LOX or naproxen (Nap) known to inhibit COX was subjected to assessment for their abilities to modulate *Se-Duox* expression (figure 4*c*). Both inhibitors significantly (*p* < 0.05) suppressed *Se-Duox* expression, with Nap being much more potent than Esc. Inhibited expression of *Se-Duox* after Nap treatment was rescued by the addition of PGE_2_ or PGD_2_. ROS level in the gut lumen was downregulated by Nap treatment (figure 4*d*). However, the suppressed ROS level caused by Nap treatment was significantly (*p* < 0.05) rescued by the addition of PGE_2_ or PGD_2_.

### 2.5. cAMP signal mediates *Se-Duox* expression

**Figure 5.**
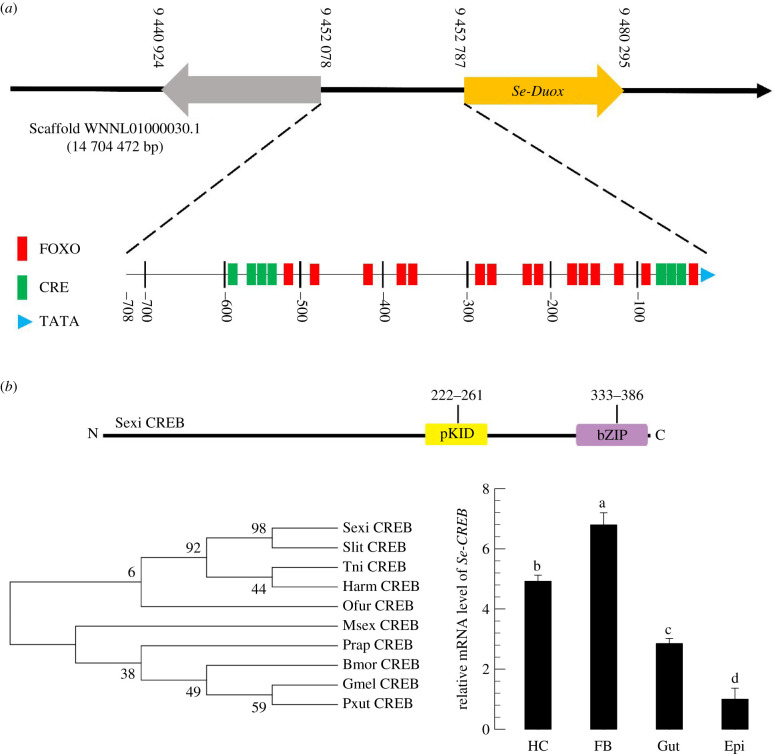
Identification of CRE and CREB associated with *Se-Duox*. (*a*) Promoter analysis of dual oxidase gene of *S. exigua* using PROMO and GPMiner programs. The upstream region from ATG start codon of *Se-Duox* was analysed for promoter components. (*b*) Functional domain analysis and phylogenetic analysis of *Se-CREB* with other lepidopteran *CREB*s based on their amino acid sequences. Domains were predicted using HMMER (https://www.ebi.ac.uk) and Pfam (http://pfam.xfam.org). Predicted domains include ‘pKID’ for phosphorylated kinase-inducible-domain and ‘bZIP’ for basic leucine zipper. The tree was generated by the neighbour-joining method using MEGA 6.0. Bootstrapping values were obtained with 1000 repetitions to support branch and clustering. Amino acid sequences were retrieved from GenBank. Accession numbers of genes are shown in electronic supplementary material, table S3. (*c*) Expression patterns in indicated tissues of L5D2 larvae, including haemocyte (HC), fat body (FB), midgut (Gut) and epidermis (Epi). A ribosomal gene, RL32, was used as a reference gene. Each treatment was replicated three times with independent tissue preparations. Different letters above standard deviation bars indicate significant differences among means at Type I error = 0.05 (LSD test).

PGR of *S. exigua* can activate cAMP secondary messenger [16]. To predict the involvement of cAMP signal pathway in modulating *Se-Duox* expression, the upstream region (708 bp) of *Se-Duox* open reading frame (ORF) was analysed to determine the presence of any cAMP-related promoter element (figure 5*a*). In addition to the TATA box, the putative promoter region contained cAMP-responsive elements (CREs) at 0 to −100 bp and −500 to −600 bp. The presence of CRE in the promoter region suggested a functional interaction with CRE-binding protein (CREB). CREB of *S. exigua* (*Se-CREB*) was predicted from the transcriptome of *S. exigua* (GenBank accession number: WNNL01000001.1) after interrogating with CREB gene of *S. litura* (NCBI GenBank accession number: XM_022964340.1). Its sequence (390 amino acids) was highly homologous to other CREB genes (figure 5*b*). This predicted *Se-CREB* was found to be expressed in different larval tissues (figure 5*c*).

**Figure 6.**
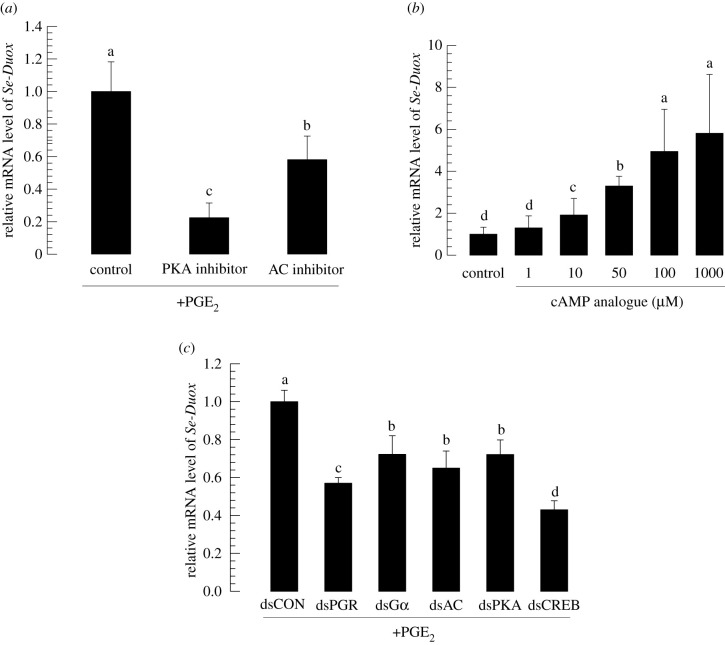
*Se-Duox* expression is regulated by PKA signalling pathway. (*a*) Inhibitory effects of PKA and AC inhibitors on *Se-Duox* expression in fifth instar larvae (L5). (*b*) Dose-dependent effect of cAMP analogue on *Se-Duox* expression in L5. For bacterial challenge and induction of Duox expression, at 8 h post-injection of inhibitors or cAMP analogue, larvae were fed with *E. coli* (2 × 10^4^ cells)-treated artificial diet for 12 h. (*c*) Downregulatory effects of RNAi specific to PKA signalling pathway components on expression levels of *Se-Duox.* At 48 h after dsRNA injection, larvae were fed with *E. coli* (2 × 10^4^ cells)-treated artificial diet for 12 h to induce Duox expression. Each treatment was replicated three times. Each replication used five larvae. Different letters indicate significant differences among means at Type I error = 0.05 (LSD test).

Specific inhibitors against adenylate cyclase (AC) producing cAMP and protein kinase A (PKA) phosphorylating proteins in response to cAMP were then used to treat larvae of *S. exigua* (figure 6*a*). Larval midguts from treated larvae expressed *Se-Duox* at significantly lower levels compared with those from control larvae without such treatment. By contrast, the administration of cAMP analogue (8-(4-chlorophenylthio)adenosine 3′,5′-cyclic monophosphate sodium salt) increased *Se-Duox* expression in a dose-dependent manner (figure 6*b*).

The role of cAMP in modulating *Se-Duox* expression suggested a signalling pathway from PGE_2_ to *Se-Duox* expression via PGR, Gαs, AC, PKA and CREB. All these five genes were downregulated after injecting their gene-specific dsRNAs (electronic supplementary material, figure S1). When *Se-Duox* expression was assessed at 72 h after each dsRNA treatment, its expression levels were significantly reduced after RNAi treatment (all five gene-specific dsRNAs) compared with those in controls (figure 6*c*).

## 3. Discussion

This study focused on gut immunity associated with ROS production. To this end, we identified a *Se-Duox* gene through bioinformatics prediction. Its expression was confirmed in all developmental stages and different larval tissues. Functional domains of Se-Duox contain two different oxidases, like other *Duox* genes [8]. Such domain prediction results suggest a pathway of ROS production in midgut epithelium. It begins at the intracellular oxidase domain by extracting two electrons from NADPH + H^+^. These electrons are then delivered to haem structures in the transmembrane domain for forming superoxide and hydrogen peroxide in the extracellular (luminal) side of the midgut. Hydrogen peroxide is then subjected to the catalytic activity of peroxidase to generate microbicidal HOCl. Mutant Duox lacking the N-terminal peroxidase domain results in severely impaired host defence system [17]. Our current study showed that RNAi specific to *Se-Duox* suppressed ROS level. Suppressed ROS level after RNAi treatment caused larvae to become susceptible to bacterial infection. This suggests that Se-Duox activity is required for immune defence but should be tightly controlled because excessive ROS production by uncontrolled Duox activity would be detrimental to insect midgut tissues. In *Drosophila*, Duox activity is activated through increased gene expression or post-translational modification [9]. Uracil derived from pathogenic bacteria can bind to G-protein-coupled receptor and trigger Gαq-PLCβ-Ca^2+^ pathway to activate MEKK1–MKK3–p38 MAPK with subsequent upregulation of Duox gene expression or increased enzymatic activity through Ca^2+^-binding domain of Duox. By contrast, Duox in another lepidopteran host, *P. xylostella*, is controlled by eicosanoid biosynthesis inhibitor [11], suggesting that there might be a cross-talk between Gαq-PLCβ-Ca^2+^ pathway and eicosanoid signalling.

*Se-Duox* expression was dependent on PLA_2_ activity. The inhibition of PLA_2_ activity suppressed *Se-Duox* expression. Such suppression in the expression of *Se-Duox* was then rescued by the addition of AA, suggesting that AA and/or its oxygenated eicosanoids could mediate gene expression of *Se-Duox*. To clarify the types of eicosanoids involved in the regulation, esculetin (leukotriene biosynthesis inhibitor) or naproxen (prostaglandin biosynthesis inhibitor) was used for treatment. The higher inhibitory activity of naproxen against *Se-Duox* expression suggested the role of PGs in mediating the gene expression. This was further supported by the rescue effect of PGE_2_ or PGD_2_. A variety of PGs other than PGE_2_ and PGD_2_ along with epoxyeicosatrienoic acids (EETs) are not assessed in this current study, and thus it remains that other types of PGs and EETs may mediate *Se-Duox* induction.

Changed PLA_2_ activities by its specific inhibitor, dexamethasone (DEX), modulated ROS production, suggesting that PGE_2_ could activate *Se-Duox* to produce ROS in the midgut. To explain this pathway, we speculate that MAPK p38 could mediate ROS production via activation of Duox gene expression in *Drosophila*'s midgut [18]. In mammalian intestine, MAPK p38 can also activate COX-2 to elevate PGE_2_ levels [19], suggesting that MAPK p38 plays a role in mediating the cross-talk between the Gαq-PLCβ-Ca^2+^ pathway and eicosanoid signal to produce ROS. Thus, we wondered how the upregulated level of PGE_2_ could activate *Se-Duox* expression.

Promoter analysis of *Se-Duox* gene indicated the presence of a CRE at the upstream region of *Se-Duox* ORF. PGR of *S. exigua* can transmit its signal using cAMP [16]. To determine the significance of CRE, this study identified a CRE-binding protein (*Se-CREB*) of *S. exigua*. Its sequence exhibited high homologies with other known CREB genes. The presence of a highly conserved serine residue was predicted to be activated after phosphorylation by PKA [20]. The leucine zipper motif in the α-helix was predicted to form a characteristic leucine zipper motif, which binds to CRE for transactivation [21]. Se-CREB was expressed in all developmental stages and different tissues of *S. exigua*. These results suggest a cAMP signal pathway of *S. exigua* from PGR to CREB via Gαs, AC and PKA signalling components.

Specific inhibitors against AC or PKA prevented the induction of *Se-Duox* expression. By contrast, the addition of cAMP analogue significantly induced the expression of *Se-Duox* in a dose-dependent manner. Thus, cAMP signalling pathway is likely to mediate *Se-Duox* expression in response to PGE_2_ in *S. exigua*. Indeed, RNAi of PGR (= PGE_2_ receptor) gene expression prevented the induction of *Se-Duox* gene. Furthermore, the cAMP signalling pathway to induce *Se-Duox* expression was supported by suppressed *Se-Duox* expression after individual RNAi treatments of its signalling components. Alternatively, calcium signalling pathway may play a crucial role in activating Duox enzyme activity [4]. Especially, Se-Duox contains EF domain suggesting its catalytic activity depending on Ca^2+^. A specific PG receptor in oenocytoi haemocytes of *S. exigua* activates calcium signalling pathway [13]. PG-Ca^2+^-Duox activity needs to be clarified in the subsequent study.

**Figure 7.**
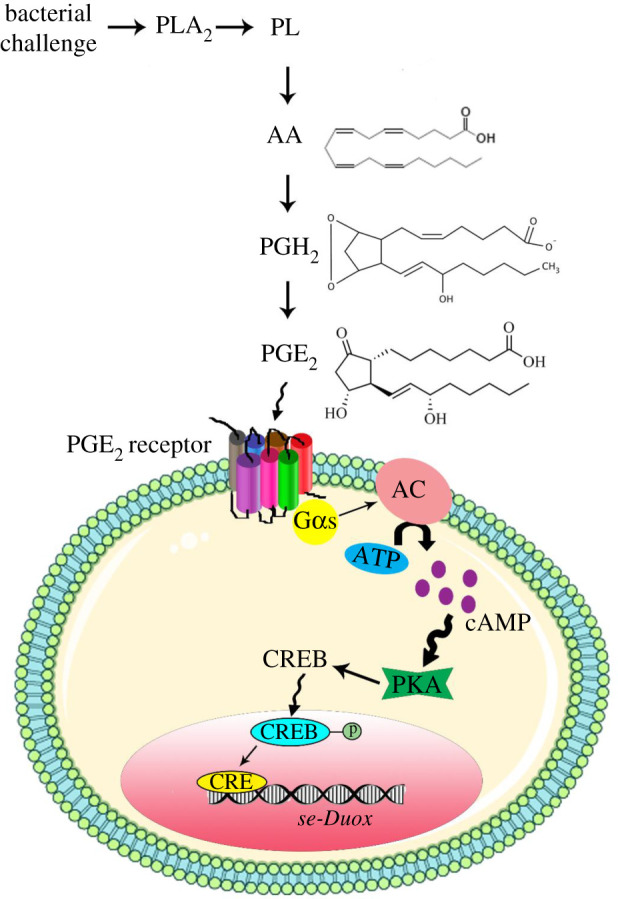
A schematic of *Se-Duox* expression under the control of PKA signalling pathway. In response to bacterial challenge, PGE_2_ receptor on the cell surface is activated. AC can increase the amount of cAMP in the cytosol which induces PKA. Induced PKA causes CREB phosphorylation and subsequent translocation to the nucleus. CREB in the nucleus acts as a transcription factor and upregulates *Se-Duox* expression.

This study showed that *Se-Duox* expression in the gut was mediated by PGE_2_ via cAMP secondary messenger (figure 7). MAPK p38 induced by pathogenic bacterial infection might induce PG production via a COX-like enzyme in *S. exigua*. A specific peroxidase called peroxynectin (Pxt) is known to mediate PG production in *S. exigua* [22]. PGE_2_, a main PG member in *S. exigua* [16], can activate the cAMP signal via its specific receptor, leading to the expression of *Se-Duox* to produce ROS in the midgut to defend microbial pathogens. PGE_2_ is produced in midguts of different insects including *Manduca sexta* and *Helicoverpa zea* (two lepidopteran insects), *Periplanata americana* (cockroach) and *Anopheles gambiae* (mosquito) [15,23]. In addition to ROS production caused by induced *Duox* expression, PGE_2_ can also mediate other gut immune responses by recruiting haemocytes to infection foci [15] and activating gene expression of antimicrobial peptides [24] in mosquitoes. All these immune mediation by PGE_2_ should be preceded by pathogen recognition in the midgut. This study suggests that uracil-based Gαq-PLCβ-Ca^2+^ recognition signal can activate eicosanoid signal pathway via p38 MAPK in *S. exigua*. Further study is needed to identify a specific p38 kinase that can activate Pxt to produce PGE_2_.

## 4. Methods

### 4.1. Insect rearing and bacterial culture

All experiments were conducted with a lepidopteran model, *S. exigua* larvae. Rearing of *S. exigua* larvae followed the method described previously [25]. Under rearing conditions, *S. exigua* underwent five larval instars (L1–L5) before pupation. *Escherichia coli* Top10A, a Gram-negative bacterium, was obtained from Invitrogen (Carlsbad, CA, USA) and cultured overnight in Luria-Bertani (LB) medium at 37°C. *Serratia marcescens*, a Gram-negative bacterium, was cultured in nutrient broth medium for 20 h at 28°C in a shaking (200 rpm) incubator. After centrifuging the cultured broth at 8000*g* for 20 min, bacterial cells were collected with 100 mM phosphate-buffered saline (PBS, pH 7.4) and used for immune challenge. *Escherichia coli* (2 × 10^4^ cells larva^−1^) and *S. marcescens* (10^5^, 10^6^ and 10^7^ colony-forming unit (CFU) ml^−1^) were used for immune challenge through feeding assays after dipping food/diet in bacterial suspensions. For each bacterial concentration, 10 test larvae were used. Each treatment was replicated three times.

### 4.2. Chemicals

Arachidonic acid (AA: 5,8,11,14-eicosatetraenoic acid), dexamethasone (DEX: (11β,16α)-9-fluoro-11,17,21-trihydroxy-16-methylpregna-1,4-diene-3), naproxen (Nap: (*S*)-(+)-6-methoxy-α-methyl-2-naphthaleneacetic acid), esculetin (Esc: 6,7-dihydroxycoumarin) and cAMP analogue (8-(4-chlorophenylthio)adenosine 3′,5′-cyclic monophosphate sodium salt) were purchased from Sigma-Aldrich Korea (Seoul, Korea). Prostaglandin D_2_ (PGD_2_: 9α,15S-dihydroxy-11-oxo-prosta-5Z,13E-dien-1-oic acid) and prostaglandin E_2_ (PGE_2_: 9-oxo-11α,15S-dihydroxy-prosta-5Z,13E-dien-1-oic acid) were purchased from Cayman Chemical (Ann Arbor, MI, USA). PKA inhibitor H89 dihydrochloride (*N*-2-3-(4-bromophenyl)-2-propenylaminoethyl-5-isoquinolinesulfonamide dihydrochloride) was purchased from Tocris (Bristol, UK). Adenylyl cyclase type V inhibitor (2-amino-7-(furanyl)-7,8-dihydro-5(6H)-quinazolinone) was purchased from Calbiochem (San Diego, CA, USA). All these chemicals were dissolved in dimethyl sulfoxide (DMSO).

### 4.3. Midgut tissue preparation

Midguts were isolated from larval instars (L1–L5), pupae (2–3 days old) and adults (2–3 days old). For L1–L2 stages, 50 individuals were used to collect a midgut sample. For L3–L4 stages, 10 individuals were used. By contrast, only one individual was used to collect a midgut sample from L5 larva, pupa or adult.

### 4.4. Bioinformatics

*Duox* sequence (NCBI GenBank accession number: NM_134871.3) of *D. melanogaster* was used to obtain its orthologue (*Se-Duox*) from *S. exigua* using whole-genome shotgun database deposited at GenBank with BlastN search engine. To localize the promoter region of *Se-Duox*, a scaffold (accession number: WNNL01000030.1) was subjected to analysis with FGENESH (http://www.softberry.com) to predict gene loci. Putative promoter (708 bp upstream from ATG start codon) of *Se-Duox* was analysed using PROMO (http://alggen.lsi.upc.es). GPMiner (http://gpminer.mbc.nctu.edu.tw) was used to identify putative transcription factor-binding sites. Protein domains of *Se-Duox* were predicted using HMMER (https://www.ebi.ac.uk) and Pfam (http://pfam.xfam.org). Phylogenetic analyses and phylogenetic tree construction with the neighbour-joining method were performed using MEGA6 and ClustalW programs. Bootstrapping values were obtained with 1000 repetitions to support branching and clustering.

### 4.5. RNA extraction and cDNA construction

Total RNAs were extracted from all developmental stages of *S. exigua* (egg stage, more than 1000 eggs were used for each extracton; L1, 30 larvae; L2, 20 larvae; L3, 10 larvae; L4, three larvae; L5, one larva; one pupa; one adult) using Trizol reagent (Invitrogen) according to the manufacturer's instruction. Different tissue samples were isolated from L5 larvae, in which fat body, midgut and epidermis were collected from five larvae as an experimental unit. Haemocytes were collected from 30 larvae per replication. Extracted RNAs were dissolved in 50 µl of diethyl pyrocarbonate-treated deionized and distilled water. First-strand cDNAs were synthesized from 1 µg of RNAs using Maxime RT PreMix (Intron Biotechnology, Seoul, Korea) containing oligo dT primers according to the manufacturer's instruction.

### 4.6. RT-PCR and RT-qPCR

Synthesized cDNAs were used as templates for PCR amplification or for constructing dsRNAs. Real-time quantitative polymerase chain reaction (qPCR) was carried out in a total volume of 20 µl consisting of 2× SYBR Green Realtime PCR Master Mix (Toyobo, Osaka, Japan), 5 mM of gene-specific forward and reverse primers and 80 ng cDNA as template. PCR amplification was performed at 95°C for 10 min as an initial heat treatment step followed by 40 cycles of 98°C for 30 s, 52°C for 30 s and 72°C for 30 s. It was then finished with a final extension step at 72°C for 7 min. As an endogenous control for constitutive expression, a stably expressed ribosomal gene, *RL32*, was assessed along with test samples. Melting curves of products were obtained to confirm amplification specificity. A comparative *C*_T_ method [26] was used to estimate relative gene expression. All experiments were replicated three times with independent samples of test stages or tissues. All primer sequences used in this study for RT-PCR and RT-qPCR are presented in electronic supplementary material, table S1.

### 4.7. dsRNA preparation and RNAi

Double-stranded RNAs (dsRNAs) specific to *Se-Duox* and other genes were prepared as described previously [11]. Briefly, a partial *Se-Duox* sequence was produced by PCR using gene-specific primers containing a T7 promoter sequence at the 5′ end (electronic supplementary material, table S1). The PCR product was used as a template to generate dsRNA using a MEGAscript RNAi kit (Ambion, Austin, TX, USA) according to the manufacturer's instruction. Sense and antisense RNA strands were synthesized using T7 RNA polymerase at 37°C for 4 h. A control dsRNA (dsCON) was also prepared by synthesizing 520 bp fragment dsRNA of CpBV302, a viral gene. The resulting dsRNA was purified and mixed with transfection reagent Metafectene PRO (Biontex, Plannegg, Germany) at a ratio of 1 : 1 (v/v) followed by incubation at 25°C for 30 min to form liposomes. One microgram of dsRNA was injected to larval haemocoel (3-day-old L4 larvae) using a microsyringe (Hamilton, Reno, NV, USA) equipped with a 26 gauge needle. At 24, 48 and 72 h post-injection (PI), RNAi efficacies were determined using RT-qPCR as described above. At 48 h PI, treated larvae were used for immune challenge and measurement of *Se-Duox* expression after an oral infection. Each treatment was replicated three times.

### 4.8. ROS measurement

ROS quantification was conducted using an OxiSelect Intracellular ROS Assay Kit (cat. no. STA-342, Cell Biolabs Inc., San Diego, CA, USA). After squeezing out contents of the dissected midguts, remaining gut tissues were then washed twice with 1 ml of PBS by repetitive resuspension followed by centrifugation at 800*g* for 5 min. Washed tissues were then suspended in TC-100 cell culture medium containing 0.1× DCFH-DA (dichlorofluorescein diacetate, 20× DCFH-DA stock, Part no. 234201) and incubated at 37°C for 30 min with gentle mix (inverting). After washing tissues three times with PBS, tissues were dissolved in 500 µl of Cell Lysis Buffer (Part no. 234203) diluted with TC100 cell culture medium and incubated at room temperature for 5 min. Cell lysate (150 µl) was then transferred to a 96-well plate and fluorescence was read at emission wavelength of 530 nm after excitation at 480 nm. For extracellular ROS measurement, DCFH-DA was mixed with midgut content and incubated at 37°C for 30 min. A calibration curve was drawn using serial dilutions of DCF (dichlorofluorescein) standard (part no. 234202) in TC-100 cell culture medium.

### 4.9. Applications of different inhibitors

All inhibitors used in this study were dissolved in DMSO at 10 µM and injected into L5 larvae (1 µl larva^−1^). At 8 h PI, treated larvae were used to estimate *Se-Duox* expression levels. For oral administration, gene expression analysis was performed at 12 h after feeding treatment along with the bacterial challenge.

### 4.10. Data analysis

All data are presented as mean ± s.e. and plotted with Sigma plot. To determine statistical differences, means were compared with Fisher's least squared difference (LSD) test and discriminated at Type I error = 0.05. Significant differences in RNAi efficiency were tested using the *t*-test of Sigma plot software v. 12.5. A *p*-value of less than 0.05 was considered statistically significant. All experiments were performed using three independent biological replicates.

## Supplementary Material

Supplementary data

## Data accessibility

This article has no additional data.
