# Supplementary material for: PGE2 upregulates gene expression of dual oxidase in a lepidopteran insect midgut via cAMP signalling pathway
Source: Open Biol. 2020 Oct 21;10(10):200197. doi: 10.1098/rsob.200197 (PMC7653354; doi:10.1098/rsob.200197)
Supplement: Supplementary data [file rsob200197supp1.docx]

***Open Biology***

**PGE_2_ up-regulates gene expression of dual oxidase in a lepidopteran insect midgut via cAMP signaling pathway**

**Seyedeh Minoo Sajjadian and Yonggyun Kim**

*Department of Plant Medicals, College of Life Sciences, Andong National University, Andong 36729, Korea*

Corresponding author: [hosanna@anu.ac.kr](mailto:hosanna@anu.ac.kr)

**Supplementary data**

**Table S1**. Primers used in this study

**Table S2**. GenBank accession numbers used for phylogenetic analysis in Fig. 1B

**Table S3**. GenBank accession numbers used for phylogenetic analysis in Fig. 5B

**Figure S1.** Effects of RNA interference (RNAi) using dsRNAs specific to components of PKA signaling pathway on *Se-Duox* expression. Larvae were injected with 1 μg of double‐stranded RNA (dsRNA) specific to (A) prostaglandin receptor (‘dsPGR’), (B) G protein αs subunit (‘dsGαs’), (C) Adenylate cyclase (‘dsAC’), (D) protein kinase A (‘dsPKA’), and (E) cAMP response element-binding protein (‘dsCREB’). Control (‘dsCON’) dsRNA treatment used dsRNA specific to CpBV302, a viral gene. Total RNAs were extracted from midguts of *S. exigua* larvae at 24, 48, and 72 h after dsRNA injection and assessed by RT‐qPCR. A ribosomal protein (RL32) gene was used as a housekeeping gene to normalize qPCR data. Each treatment consisted of three independent replications. Each treatment was replicated three times. Each replication used 5 larvae. Different letters indicate significant differences among means at Type I error = 0.05 (LSD test).

**Table S1.** Primers used in this study

| Primer | Uses | Sequence (5ʹ - 3ʹ) | Annealing temperature (ºC) | Expected size of PCR product (bp) |
| --- | --- | --- | --- | --- |
| Se-Duox | RT-PCR  RT-qPCR | AGGATCGTTATCGCTAGTTTACAG | 52 | 308 |
|  |  | GCCATCCCTAATAGGACTTCTTC |  |  |
| T7_Se-Duox | RNAi | TAATACGACTCACTATAGGGAGAAGGATCGTTATCGCTAGTTTACAG | 52 | 308 |
|  |  | TAATACGACTCACTATAGGGAGAGCCATCCCTAATAGGACTTCTTC |  |  |
| Se-PGR | RT-PCR  RT-qPCR | CTCACAAGGCCCTTCCTCTAC | 52 | 240 |
|  |  | GTTGCAGTACACCACCACCA |  |  |
| T7_Se-PGR | RNAi | TAATACGACTCACTATAGGGAGACTCACAAGGCCCTTCCTCTAC | 52 | 240 |
|  |  | TAATACGACTCACTATAGGGAGAGTTGCAGTACACCACCACCA |  |  |
| Se-Ga | RT-PCR  RT-qPCR | GGAGAAGATCGAGGACATCAAG | 52 | 327 |
|  |  | TGCACCTGATCCAGGAAATAC |  |  |
| T7_ Se-Ga | RNAi | TAATACGACTCACTATAGGGAGAGGAGAAGATCGAGGACATCAAG | 52 | 327 |
|  |  | TAATACGACTCACTATAGGGAGATGCACCTGATCCAGGAAATAC |  |  |
| Se-AC | RT-PCR  RT-qPCR | GATGGTGCACACAATGATGCC | 52 | 306 |
|  |  | GGTATGACAGAGAGGAGCAATC |  |  |
| T7_Se-Ac | RNAi | TAATACGACTCACTATAGGGAGAGATGGTGCACACAATGATGCC | 52 | 306 |
|  |  | TAATACGACTCACTATAGGGAGAGGTATGACAGAGAGGAGCAATC |  |  |
| Se-PKA | RT-PCR  RT-qPCR | TACGCGGTCGAGTTCTTCAC | 52 | 445 |
|  |  | CCACCTTCTCGATGCGATCA |  |  |
| T7_Se-PKA | RNAi | TAATACGACTCACTATAGGGAGATACGCGGTCGAGTTCTTCAC | 52 | 445 |
|  |  | TAATACGACTCACTATAGGGAGACCACCTTCTCGATGCGATCA |  |  |
| Se-CREB | RT-PCR  RT-qPCR | AAGTACCGGGAAATGCTCAC | 52 | 410 |
|  |  | GCTGGACCTCATTCTGTCTTT |  |  |
| T7_Se-CREB | RNAi | TAATACGACTCACTATAGGGAGAAAGTACCGGGAAATGCTCAC | 52 | 410 |
|  |  | TAATACGACTCACTATAGGGAGAGCTGGACCTCATTCTGTCTTT |  |  |
| dsCON | RNAi | CCCACTAGTGTCCTCATCACCTCCTCAAAC | 52 | 520 |
|  |  | CCCAAGCTTCAGAGTCACCGTTGCAAGTA |  |  |
| RL32 | RT-PCR  RT-qPCR | ATGCCCAACATTGGTTACGG | 52 | 270 |
|  |  | TTCGTTCTCCTGGCTGCGGA |  |  |

*Underlined sequences represent T7 sequence

**Table S2.** GenBank accession numbers used for phylogenetic analysis in Fig. 1B

| GenBank accession number | Acronym | GenBank accession number | Acronym |
| --- | --- | --- | --- |
| XP_021700460.1 | Aaeg duox | XP_022920959.1 | Otau duox |
| ETN58940.1 | Adar duox | PNF38268.1 | Csec duox |
| AKS43593.1 | Bdoe duox | XP_021932850.1 | Znev duox |
| XP_004533990.1 | Ccap duox | XP_029676591.1 | Fexs duox |
| XP_026843273.1 | Dper duox | XP_011156000.1 | Sinv duox |
| KNC33589.1 | Lcup duox | XP_026735041.1 | Tni duox |
| XP_011189298.1 | Zcuc duox | XP_022813998.1 | Slit duox |
| XP_018323658.1 | Apla duox | XP_011558844.1 | Pxyl duox |
| XP_018563303.1 | Agla duox | KPJ08497.1 | Pmac duox |
| XP_019766183.1 | Dpon duox | XP_028176585.1 | Ofur duox |
| XP_028130484.1 | Dvir duox | XP_025856993.1 | Harm duox |
| XM_023156626.1 | Ldec duox |  |  |

**Table S3.** GenBank accession numbers used for phylogenetic analysis in Fig. 5B

| GenBank accession number | Acronym | GenBank accession number | Acronym |
| --- | --- | --- | --- |
| XP_022820104.1 | Slit CREB | XP_022114368.1 | Prap CREB |
| XP_021181206.1 | Harm CREB | XP_021202733.1 | Bmor CREB |
| XP_026746772.1 | Tni CREB | XP_026757619.1 | Gmel CREB |
| XP_030031351.1 | Msex CREB | XP_028177052.1 | Ofur CREB |
| XP_013182292.1 | Pxut CREB | WNNL01000001.1 | Sexi CREB |

**(A) (B)**

**(C) (D)**

**(E)**

**Fig. S1**
